# Residual Stress Distribution in a Copper-Aluminum Multifilament Composite Fabricated by Rotary Swaging

**DOI:** 10.3390/ma16052102

**Published:** 2023-03-05

**Authors:** David Canelo-Yubero, Radim Kocich, Jan Šaroun, Pavel Strunz

**Affiliations:** 1 Neutron Physics Department, Nuclear Physics Institute of the CAS, 25068 Řež, Czech Republic; 2 Institute of Materials Physics, Helmholtz-Zentrum Hereon, Max-Planck-Straße 1, 21502 Geesthacht, Germany; 3 Faculty of Materials Science and Technology, VŠB–Technical University of Ostrava, 17. Listopadu 15, 70833 Ostrava, Czech Republic

**Keywords:** composite, aluminum, copper, severe plastic deformation, rotary swaging, residual stress, neutron diffraction, finite element simulation, von Mises

## Abstract

Rotary swaging is a promising technique for the fabrication of clad Cu/Al composites. Residual stresses appearing during the processing of a special arrangement of Al filaments within the Cu matrix and the influence of the bar reversal between the passes were studied by (i) neutron diffraction using a novel evaluation procedure for pseudo-strain correction and (ii) a finite element method simulation. The initial study of the stress differences in the Cu phase allowed us to infer that the stresses around the central Al filament are hydrostatic when the sample is reversed during the passes. This fact enabled the calculation of the stress-free reference and, consequently, the analysis of the hydrostatic and deviatoric components. Finally, the stresses with the von Mises relation were calculated. Hydrostatic stresses (far from the filaments) and axial deviatoric stresses are zero or compressive for both reversed and non-reversed samples. The reversal of the bar direction slightly changes the overall state within the region of high density of Al filaments, where hydrostatic stresses tend to be tensile, but it seems to be advantageous for avoiding plastification in the regions without Al wires. The finite element analysis revealed the presence of shear stresses; nevertheless, stresses calculated with the von Mises relation show similar trends in the simulation and in the neutron measurements. Microstresses are suggested as a possible reason for the large width of the neutron diffraction peak in the measurement of the radial direction.

## 1. Introduction

In applications where contradictory material properties are required, the use of single-phase materials may not be optimal. In composites, the microstructure and properties can be adapted for special functionalities. Therefore, composites are increasingly the focus of technologists thanks to their capability to achieve advantageous properties in two or more fields (e.g., thermal and electrical) [1,2], and possibly to reduce the price of the components at the same time.

Layered composites, also referred to as clad composites, are innovative materials consisting of two or more different metals bonded at mutual interfaces. Similarly, multifilament composites can be produced with the filaments of one metallic material embedded in the matrix of the other metal. Such composites enable a combination of various properties (e.g., corrosion and wear resistance, thermal and electric conductivity, acoustic damping and energy absorption, and magnetic properties) in the final component. They are used in the transport industry (aerospace, automotive, and marine [3,4]). They are used for coils of superconducting magnets as well [5] in thermal engineering [6] and in medicine [7]. They also have applicability for signal transmission as well as electric power transmission [8,9].

There is an ongoing search for novel composite materials for the replacement/complement of relatively expensive and heavy copper conductors. The addition of aluminum is intended to reduce weight and improve the properties of the copper components originally used in electrical engineering. The main advantage of Cu/Al composites is that they combine the high strength and excellent electrical conductivity of Cu with the light weight and low cost of Al. Cu-based clad conductors are efficient for applications using alternating current (AC) thanks to the so-called “skin effect”. Further, Cu-based multifilament conductors are promising for their use in direct current (DC) and low-frequency AC applications.

There is certainly room for improvement in the properties of Cu/Al clad and multifilament composites. For example, uniquely sequenced composite wires led to improvement of the microstructure after rotary swaging [10]. Further, Kim and Hong [11] produced a rolled Cu/Al/Cu composite sheet to improve the mechanical properties of the individual rolled Cu and Al. Recently, Jin et al. [12] produced Al/Cu bimetal tubes via the tube spinning process. This indicates that not only the selection of individual materials but also appropriate production technology and its optimization are the focus of researchers’ attention. Alternative composite production routes by conventional [13,14,15,16,17,18,19] or nonconventional methods of severe plastic deformation have been developed.

The main advantage of the non-conventional severe plastic deformation methods (e.g., high-pressure torsion-HPT, equal channel angular pressing-ECAP and its modifications, or friction stir-based methods) is their positive effect on the microstructure of the processed materials. They cause the formation of ultra-fine grains by imposing severe shear strain without the need for processing at elevated temperatures.

Rotary swaging, in which high-frequency strokes of the swaging dies impose increments of strain, is favorable for the fabrication of clad composites as well. A primary advantage of rotary swaging is the relatively easy production of axi-symmetrical composites of virtually unlimited length, exhibiting fine grains and enhanced mechanical properties [20]. This intensive plastic deformation technique can be used to produce and—at the same time—improve the properties of clad or multifilament composites.

Manufacturing technology for clad and multifilament composites is selected with the aim of ensuring the highest possible quality of mutual bonding between the individual components. For example, when produced under cold conditions, clad composites are prone to local debonding at the interfaces [20,21]. On the other hand, when formed under hot conditions, the elevated temperature can induce the formation and development of intermetallic layers and affect the quality of the mutual bonding [22]. Other processing parameters, such as the number of passes or reversal between the passes, can affect the properties of the final component as well. Therefore, the processing parameters have to be optimized.

Accumulation of residual stresses depending on the processing parameters can occur in rotary swaged bars. Residual stress can develop during the lowering of the diameter of the original bar as a result of various influencing factors (e.g., non-uniform heating/cooling or the inhomogeneous distribution of the imposed strain [23]). As residual stresses (i) play a role during static/dynamic loading, (ii) affect mechanical properties, and (iii) induce premature failure, understanding them and their possible suppression by selecting an appropriate processing route is of utmost importance.

One of the techniques beneficially used for residual stress determination in polycrystalline metallic components is neutron diffraction [24]. The large penetration of thermal neutrons into most metallic materials enables nondestructive measurements of elastic strains within the bulk of large polycrystalline components. Although copper matrix has a relatively high attenuation for neutrons, a path length through the sample of up to 40 mm is still possible even with a medium flux neutron source. As a consequence of the use of neutrons, the signal is averaged over a relatively large volume and, therefore, large grain size and possible local artifacts are thus minimized. On the other hand, pseudo-strains [25] can appear not only near the surface but also near the interfaces between the individual metals inside the investigated component. This effect has to be considered and represents a challenge for neutron diffraction data evaluation.

The primary research aim is to optimize Cu/Al multifilament composite production by rotary swaging. The achievement of advantageous properties on one side, and the economy of production on the other side have to be considered. This particular study focused on the determination of residual stresses in the rotary swaged bars representing a proxy of Cu/Al multifilament composites as a function of the processing route. The output will help to design a combination of parameters of the intensive plastic deformation for the optimum performance of the final product.

## 2. Materials and Methods

### 2.1. Cu/Al Composite

The initial materials used for the fabrication of the multifilament Cu/Al composite were commercially pure Cu (Cu with 0.002% O, 0.015% P, and 0.002% Zn) and electro-conductive commercially pure Al (Al with 0.20% Si, 0.25% Fe, and 0.05% Cu). The initial outer diameter of the Cu billet was 50 mm, and the diameter of the Al inserts was 10 mm. The Cu/Al volume ratio within the composite was 80:20.

**Figure 1:**
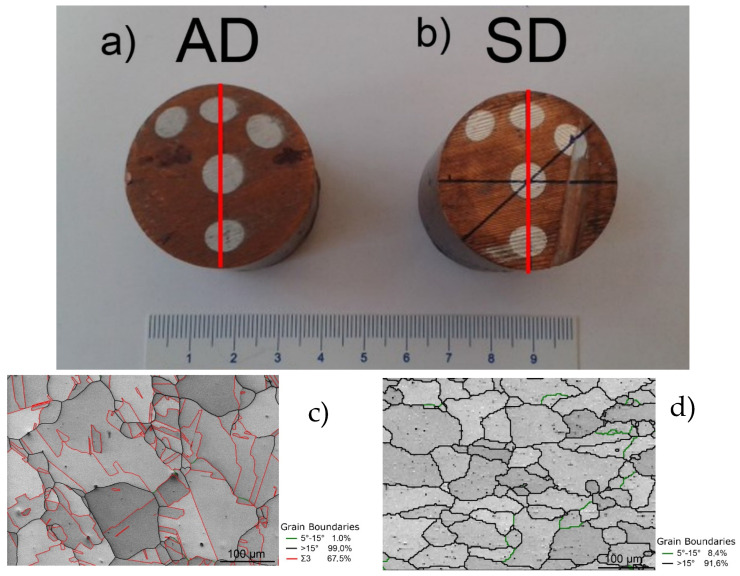
Photo of the two samples used in the study: (**a**) sample with reversal of the bar direction between the passes, denoted AD (the alternating direction) and (**b**) sample without reversal of the bar direction between the passes, denoted SD (the same direction). The red lines indicate the sample symmetry axes on the cross-section. The initial microstructures are shown in (**c**) for the Cu matrix and in (**d**) for the Al filaments. The annealing twins are depicted in red, HAGB in black, and LAGB in green.

The original bar was rotary swaged to 35 mm diameter. The deformation was carried out at room temperature (25 °C). The composites were swaged down in three swaging passes and the reduction ratio φ, calculated as ln(S_0_/S_n_), where S_0_ and S_n_ are the cross-sectional areas at the input and output of swaging dies, respectively, was 0.71. A special arrangement of the Al filaments was used in order to see the effect of various configurations within the sample on residual stresses. The measured cylindrical samples of approximately 37 mm in height are shown in Figure 1a,b. The initial microstructures of Cu and Al are shown in Figure 1c,d, respectively. The images were acquired via SEM-EBSD measurements on a Lyra 3 XMU scanning electron microscope (SEM, Tescan, Brno, Czech Republic). In the images, high angle grain boundaries (HAGB) defined with misorientation angles > 15° are depicted in black, low angle grain boundaries (LAGB) defined as misorientation angles 0° < θ < 15° are shown in green, and the coincidence site lattice (CSL) boundaries characterizing <111> 60° annealing twins are depicted in red. The composite matrix, i.e., Cu, was characterized by grains with an average size of 22.7 μm (measured as equivalent circle diameter), although the maximum grain size reached up to 160 μm. The structure also exhibited the notable presence of twins (Figure 1c). On the contrary, the original structure of the Al filaments did not show the presence of twins but a higher fraction of LAGB (Figure 1d). The average grain size within the Al was 38.5 μm, with the largest grains reaching up to 115 μm.

Two processing routes were used for the sample fabrication: with reversal of the bar direction between the passes, denoted AD (the alternating direction), and without reversal of the bar direction between the passes, denoted SD (the same direction).

### 2.2. Plastometric Tests

**Figure 2:**
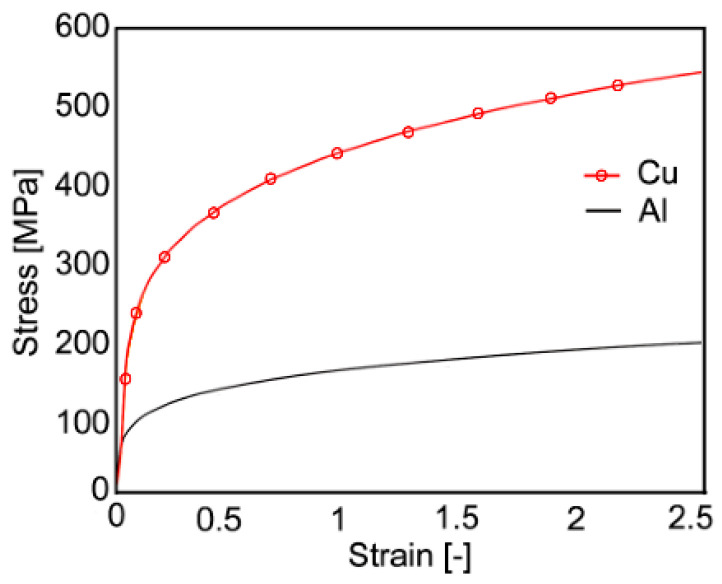
Stress-strain curves of the original commercially pure Cu and Al (torsion test).

The residual stress distribution within the swaged composites can be evaluated with the use of finite element modeling (FEM). For this purpose, torsion plastometric tests were performed to develop a reliable numerical model. Torsion tests are suitable to simulate the plastic flow during rotary swaging, as the shear strain is the dominant deformation mechanism for both metals. The stress-strain curves, depicted in Figure 2 for Cu and Al, were acquired using a servo-hydraulic torsion plastometer (SETARAM device) at room temperature and at a strain rate of 1 s^−1^.

### 2.3. Neutron Diffraction Experiments

The residual stress is the stress that remains after processing in a component that is stationary and at equilibrium with its surroundings. The residual stresses are categorized by the scale over which they self-equilibrate [26]. This paper deals with type I stresses, i.e., macrostresses.

An elastic strain measurement using neutron diffraction is based on the determination of the *d_hkl_* interplanar distance for the selected crystallographic plane. The strain was in this way determined in the processed bars using the SPN-100 neutron diffractometer of CANAM NPL infrastructure [27], installed at the LVR-15 research reactor neutron source in Řež, CZ. A sketch of the instrument is shown elsewhere [28]. This experimental facility is dedicated to the mapping of bulk-averaged residual strains in polycrystalline materials. The SPN-100 instrument is equipped with a horizontally bent Si monochromator (curvature 0.165 m^−1^), a radial collimator, and a position-sensitive detector for fast recording of diffraction patterns. A neutron wavelength of *λ* = 0.213 nm and the Cu-111 reflection were selected for the experiment, leading to a *2θ* diffraction angle of approximately 61.4°.

The incident beam was defined by 5 × 24 mm^2^ and 5 × 5 mm^2^ cadmium slits for the hoop/radial and for the axial strain measurements, respectively. The slits were positioned at 75 mm in front of the gauge volume center (instrumental sample axis). A larger height of the gauge volume was chosen for the hoop and radial directions due to the low diffraction intensity in these directions. This is a consequence of the preferred grain orientation (texture) formed during rotary swaging.

**Figure 3:**
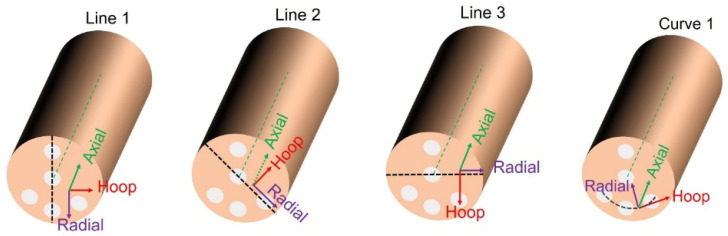
Sketch of the samples indicating the measured lines and three orthogonal strain directions: axial (1), radial (2), and hoop (3). The sketched lines were not measured at the surface but inside the sample, approximately in the middle of the length of the sample bar.

An oscillating radial collimator of 2 mm effective width was used for the diffracted beam for all the strain types. The *2θ* angle determined in the diffraction experiment was averaged over the elongated instrumental gauge volume of ≈274 mm^3^ and ≈57 mm^3^ for the hoop/radial and for the axial strains, respectively. To record the diffraction peaks for all three directions, three different geometrical arrangements of the examined sample with respect to the scattering vector were used. Samples were positioned with high accuracy by using a robotic arm. The lines along which the scans were carried out are indicated in Figure 3. The peaks from Cu-111 reflection were collected during the line scans through the sample cross section approximately in the middle of the length of the cylindrical sample.

**Figure 4:**
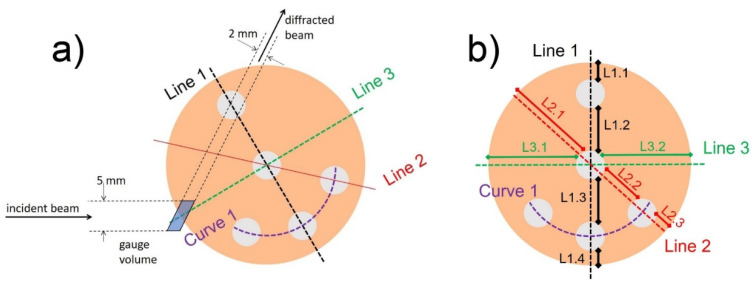
(**a**) Detailed sketch of the sample cross section indicating the measured lines and the gauge volume used for sample scanning of hoop and radial strains. (**b**) Zones defined between the Al wires are indicated with two numbers where the first corresponds to the scanned line and the second to the interval, both preceded by L(line).

A detailed sketch of the sample cross section indicating the measured lines, position of the incident and diffracted beam for the hoop and the radial strain components measurement, and the gauge volume used for sample scanning can be seen in Figure 4a. The different regions delimited by the Al wires are also numbered (see Figure 4b) for further simple identification/analysis: they are identified by L(ine) and two numbers with the first corresponding to the measured line (1, 2, or 3) and the second to the interval of the line (function of the number of Al wires in that line).

### 2.4. Strain and Stress Analysis

Bulk-averaged elastic strain measurement using neutron diffraction is based on the *d_hk_*_l_ interplanar distance determination of the selected crystallographic plane with Miller indices *hkl* according to the following expression (see [24], p. 150):

(1) $$\varepsilon_{1}^{hkl}=\frac{d_{hkl}-d_{0,hkl}}{d_{0,hkl}}$$

where *d_hkl_* and *d*_0,*hkl*_ are the interplanar distances measured over the gauge volume for the sample and for the stress-free reference, respectively. They are determined with help of Bragg’s law [29], i.e., from the measured angular peak positions, *2θ_hkl_*. The lower index at *ε* denotes the normal strain component in Voigt notation, which is parallel to the diffraction vector. Assuming an isotropic material, the residual stresses in one direction can be then determined from the lattice strains measured in three normal directions by means of the generalized Hook’s law as (see [24], p. 207)

(2) $$\sigma_{1}=\frac{E}{1+\nu }\varepsilon_{1}+\frac{\nu \;E}{\left(1+\nu \right)\left(1-2\nu \right)}\left(\varepsilon_{1}+\varepsilon_{2}+\varepsilon_{3}\right)$$

where *ε*_1_, *ε*_2_, and *ε*_3_ are the experimentally measured strains for the *hkl* crystallographic plane, and *E* and *ν* are the Young modulus and Poisson’s ratio, respectively, for the selected crystallographic plane. The *hkl* indexing is omitted for clarity of the expression. Corresponding relations for the 2nd and 3rd stress components are obtained by permutations of 1, 2, and 3 indices.

The elastic constants based on the Kröner model [30] for the Cu-111 were assumed to be *E* = 159 MPa and *υ* = 0.31 (see [24], p. 239). It should be noted that the expression (2) applies to three orthogonal directions regardless of whether they are or not principal directions. 

### 2.5. FEM Analysis

The deformation behavior of the Cu/Al composite during the first pass was numerically simulated using an elastic-plastic model defined by the Newton-Raphson convergent algorithm. The mesh of the cladded billet consisted of tetrahedral elements and involved 120.254 nodes, while the swaging dies were considered to be rigid. An automatic re-meshing was used as it was expected that severe shear deformations would occur during the simulated swaging process.

The experimental stress-strain curves depicted above were implemented into the material flow stress database of the software to enable the computation of the Haensel-Spittel relation (Equation (3)), which was further used for the characterization of the material behavior during swaging:

(3) $$\sigma_{f}=Ae^{m_{1}T}\varepsilon^{m_{2}}{\dot{\varepsilon }}^{m_{3}}\varepsilon^{\frac{m_{4}}{\varepsilon }}{\left(1+\varepsilon \right)}^{m_{5}T}\varepsilon^{m_{7}\varepsilon }{\dot{\varepsilon }}^{m_{8}T}T^{m_{9}}$$

where *ε* is the equivalent von Mises strain, *T* is the deformation temperature given in degrees centigrade, $\dot{\varepsilon }$ is the equivalent von Mises strain rate, *m_1_* and *m_9_* define the material sensitivity to *T*, *m_5_* is the term coupling *T* and strain (*ε*), *m_8_* is the term coupling *T* and $\dot{\varepsilon }$, *m_2_*, *m_4_*, and *m_7_* define the material sensitivity to *ε*, *m_3_* depends on the material sensitivity to $\dot{\varepsilon }$, and *A* is a multiplication factor. The dimensions of *m* parameters are always such that the exponent is dimensionless. The values of the used coefficients for Cu were 411.19 MPa, −0.00121, 0.21554, 0.01472, −0.00935, respectively, and *m*_5_ ÷ *m*_8_ was 0. The values of the coefficients for Al were 151.323 MPa, −0.00253, 0.21142, 0.03177, −0.00654, respectively, and *m*_5_ ÷ *m*_8_ was 0.

The boundary conditions were defined at a temperature of 25 °C, and the values of the parameters necessary to characterize the behavior of Cu and Al are detailed in Table 1.

## 3. Results

### 3.1. Sample Model, Measurement Geometry, and Simulation of Pseudo-Strain

The sample scans were used for the determination of the lattice strain distribution across the bar sections from which the residual stresses can be calculated by means of Equation (2). Nevertheless, before performing this step, an assessment and removal of pseudo-strains had to be carried out. Pseudo-strains (p. 344 [24,25]) in neutron diffraction data appear as spurious Bragg peak shifts in cases when the gauge volume is not fully immersed in the measured phase of the material, or if the scattering probability varies significantly across the gauge volume due to, for example, texture gradients or strong absorption. Therefore, pseudo-strain can appear in the points measured near the sample surface and also nearby the interfaces inside the sample.

Monte Carlo neutron ray-tracing simulation of the instrument [31] allowed us to determine the sampling distribution represented by a list of scattering events that the instrument can register for a given Bragg reflection. Each event is associated with spatial coordinates, incident and final wave vectors, and the apparent peak shift that the instrument would register for a given ray. The event list thus describes both the sampling distribution as well as the distribution of pseudo-strains.

**Figure 5:**
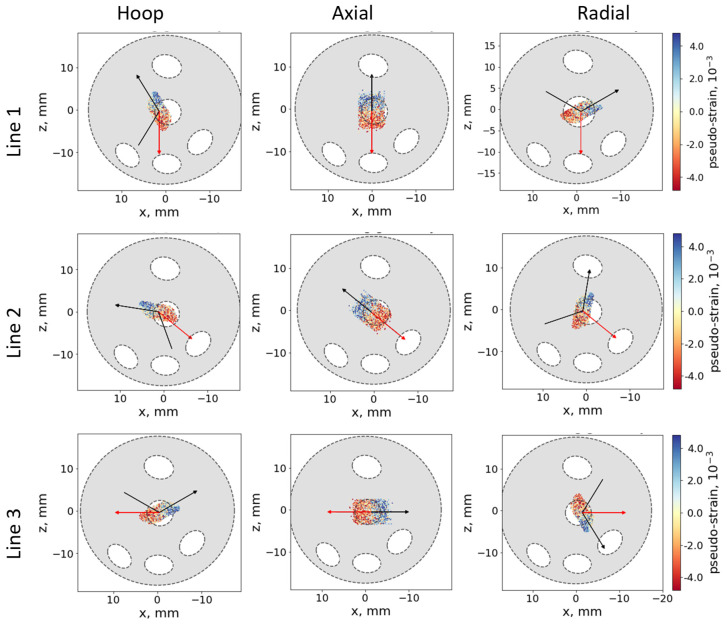
Measurement geometry (AD sample) for Line 1, Line 2, and Line 3 for hoop, axial, and radial strains. The scan direction is shown with a red arrow and the neutron beam with black arrows. The colored spots show the distribution of the simulated scattering points and the associated pseudo-strain (see the color scale).

The list of 3000 simulated scattering events representing the sampling distribution was used to carry out the convolution with the sample geometry, considering the local material properties: intrinsic lattice strain, absorption, and distribution of the scattering probability. This procedure yielded the pseudo-strain and intensity distribution along the scan, which were further used for the correction of the observed lattice strains. The Al wires were defined in the simulation model as elliptic cylinders (see Figure 1) co-axial with the sample axis. The positions and dimensions of the wires were determined from the surface images and further refined by comparison with the distribution of the scattered intensity. The Al phase was treated as a void, which is justified by the low neutron beam attenuation in Al. As an example, Figure 5 shows the simulation model of the AD sample, with the scan direction (red arrow) and neutron beams (black arrows). The colored points represent the simulated scattering events with associated pseudo-strains (see the color scale). The ray-tracing simulation was done using the program SIMRES [31]. The convolution of the sampling events with the sample properties and fitting of the intrinsic scattering probability and lattice strain distributions was done using the package STRESSFIT [32].

The evaluation of the experimental data is further explained by the example of a single scan for the radial strain direction, i.e., the scan Line 1 in sample AD.

**Figure 6:**
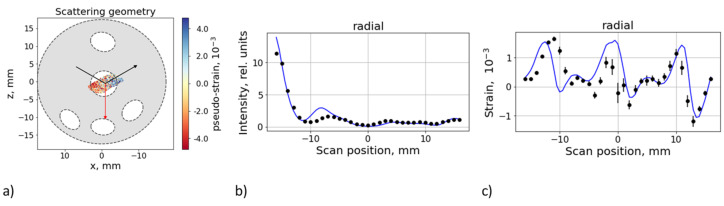
The measurement configuration is shown on the left (**a**). Example of the simulated pseudo-intensity (**b**) and pseudo-strain (**c**), compared with the experimental data for the AD sample, scan along line 1 in radial geometry.

An example of the simulated pseudo-intensity and pseudo-strain (i.e., the simulation assuming homogeneous, stress-free, and isotropic material) is shown in Figure 6. The difference between the simulated and measured strains should be ideally interpreted as the effect of intrinsic (lattice) strain. However, it is also affected by other effects:

1. Irregularity of Al wires geometry.
2. Variation of the scattering intensity due to texture gradients.

### 3.2. Fitting of Intrinsic Intensities

**Figure 7:**
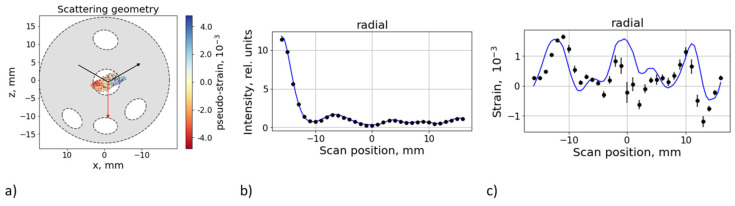
As in Figure 6, but accounting for the fitted distribution of the intrinsic scattering intensity. The measurement configuration is shown in (**a**), and the fitted intrinsic intensity distribution is shown in (**b**). The variation of simulated strain (**c**, blue line) is due to the pseudo-strain effects only; the intrinsic strain was set to zero.

Texture gradients are assumed to contribute to the variation of the scattering intensity on a scale comparable with the gauge volume size. It may give rise to additional pseudo-strains. To compensate for this effect, the distribution of the intrinsic scattering intensity along the scan was fitted to the measured intensities for each scan. This distribution was modeled by a set of 16 fixed nodes equally distributed along the scan range of (−17.5; +17.5) mm, while the intensity at each node was taken as a free parameter. Values between the nodes were calculated by the cubic spline interpolation. An example of the same scan as in the previous subsection is shown in Figure 7. The variation of the fitted intensity is partly due to texture gradients, but it is also affected by the irregularity of the wire geometry and other misalignment effects, which could not be considered in the simulations.

### 3.3. Evaluation of the Lattice Strain Distribution

**Figure 8:**
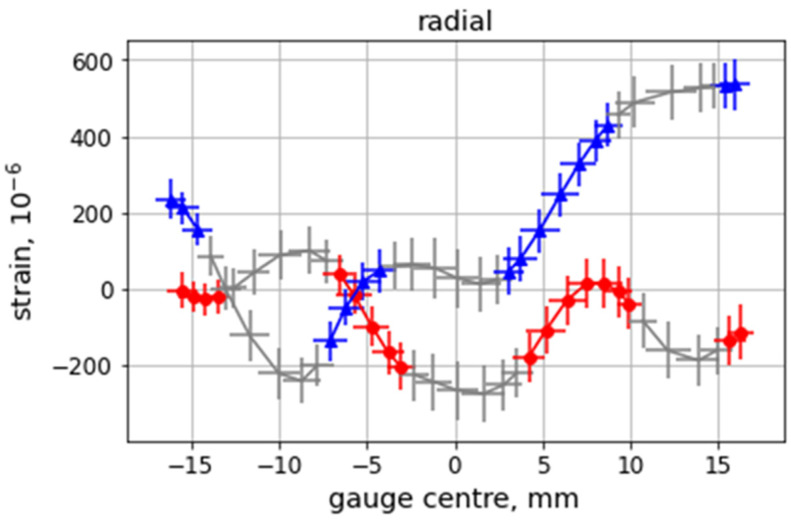
Evaluated intrinsic strain distribution by fitting the un-smeared strain distribution in the radial direction for the AD (Sample 1) and SD (Sample 2) specimens. The grey part corresponds to positions where the gauge center passes through an Al wire. Horizontal error bars indicate the size of the sampling volume along the scan direction, the vertical error bars correspond to the standard errors of the least squares fitting procedure.

The difference between the simulated pseudo-strain and measured strain (Figure 7c) can be interpreted as the lattice strain distribution, but still smeared by the spatial resolution (given by the size of the sampling volume). The evaluation was therefore undertaken by fitting the intrinsic (un-smeared) strain distribution to the measured data (see Figure 8), using the same free distribution model as for the fitting of intensities. The statistics of neutron counting in both the experiment and simulation were sufficient for gauge positions outside the Al wires. In the areas where the gauge center crosses the Al wires, the statistical errors become too high due to the low intensity (number of contributing scattering events) and the center of mass of the actual sampling is not well defined. Data from these areas must therefore be excluded. Consequently, the fitting results become unstable and prone to overfitting effects, which leads to un-physical solutions (i.e., diverging strain). To overcome this problem, a regularization technique imposing minimization of the gradient of the strain distribution was applied. Figure 8 shows an example of the fitting results with the excluded areas marked in grey. It should be noted that the regularization leads to an underestimation of the fitting errors, especially in these excluded areas.

For the strain data, the gauge center is evaluated as the center-of-mass (CoM), i.e., the mean position of the sampling events is weighed by the scattering probability and the absorption factor, which contribute to the signal in a given sampling position. A difficulty arises when calculating the CoM positions for the residual stress because the CoM differs for different strain components even if measured at the same nominal sample positions. When calculating the residual stresses, the gauge center is, therefore, calculated as the average of CoM for the three strain components. The strain values at these positions are then determined by linear interpolation.

### 3.4. d_0_ Problem and Stress Differences

The original copper powder was not available as a stress-free reference. Further, as Al is a weakly scattering matter, it would have required acquisition times significantly longer than the ones already used to determine the strains in the Cu matrix. Therefore, the strains in Al were not measured with the consequence that the equilibrium condition for the determination of the Cu stress-free reference cannot be used initially without further assumptions.

The solution proposed to overcome this issue is based on the evaluation of stress differences, which (see Equation (2)) are defined as

(4) $$\sigma_{1}-\sigma_{2}=\sigma_{axial}-\sigma_{radial}=\frac{E}{1+\nu }(\varepsilon_{axial}-\varepsilon_{radial})$$

(5) $$\sigma_{1}-\sigma_{3}=\sigma_{axial}-\sigma_{hoop}=\frac{E}{1+\nu }(\varepsilon_{axial}-\varepsilon_{hoop})$$

(6) $$\sigma_{2}-\sigma_{3}=\sigma_{radial}-\sigma_{hoop}=\frac{E}{1+\nu }(\varepsilon_{radial}-\varepsilon_{hoop})$$

**Figure 9:**
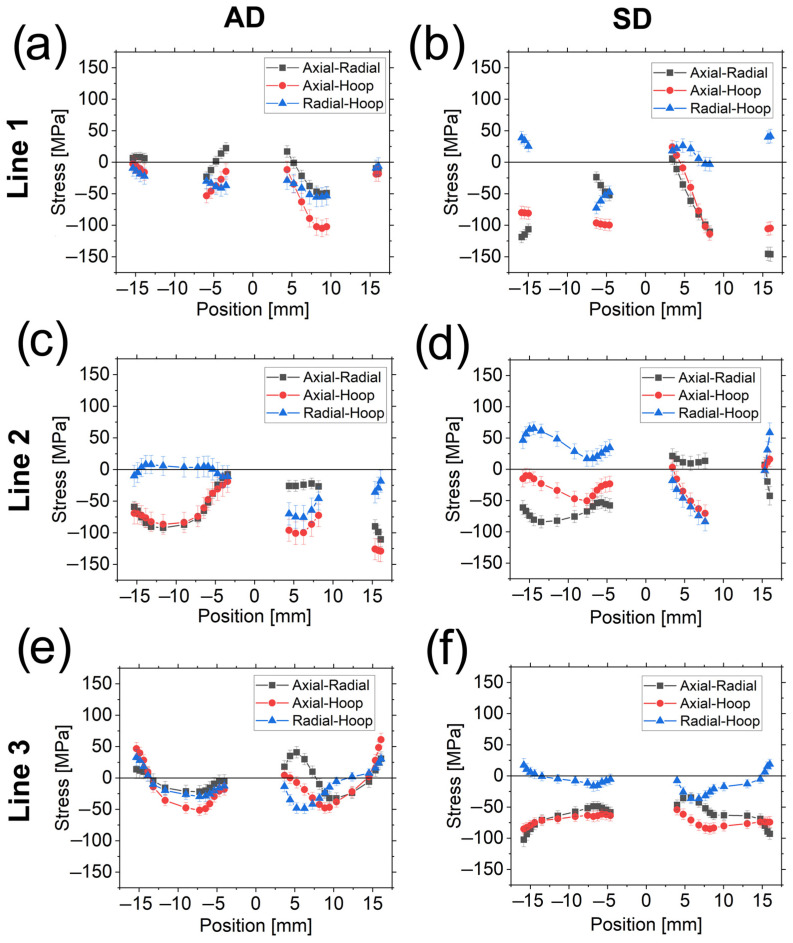
Stress differences for the AD sample (**a**,**c**,**e**) and SD sample (**b**,**d**,**f**) in Line 1 (**top**), Line 2 (**center**), and Line 3 (**bottom**).

The stress differences are shown in Figure 9 for the AD (a, c, and e) and SD (b, d, and f) samples for the scanned Line 1 (top), Line 2 (middle), and Line 3 (bottom). The error bars shown in Figure 9 and in subsequent figures in this article showing residual stresses are produced by standard error propagation rules from the fitted strains. The approximate coincidence of *σ_axial_−σ_radial_*, *σ_axial_−σ_hoop_,* and *σ_radial_−σ_hoop_* through L1.1, L1.4, and in L2.1, L3.1, and L3.2 around the central Al wire for the AD sample is remarkable. In contrast, for the SD sample, stress differences are only equal in L1.3 and roughly in the L2.2 segment around the central wire, and in L2.3 at the interface Cu-Al.

*σ_axial_−σ_radial_* can be considered equal to ≈0 MPa through L2.2 (except around the central Al wire) for the SD sample while it is shifted to −25 MPa in the same region for the AD sample.

*σ_radial_−σ_hoop_* equals to ≈0 MPa in L2.1 for the AD specimen (leading to a coincidence of *σ_axial_−σ_radial_* and *σ_axial_−σ_hoop_*). For the SD sample, *σ_radial_−σ_hoop_* roughly equals to 0 MPa in L3.1 although L3.2 seems to be an extension of the first trend showing a symmetric behavior around the axis (i.e., the position of 0 mm) roughly tilted with respect to a vertical line in the diagram.

This roughly symmetric behavior in Line 3 for the SD specimen could also be expected for the AD due to the absence of Al wires in this region (except at the center). Nevertheless, this trend is only observed close to the outer surface, although with *σ_axial_−σ_radial_* and *σ_axial_−σ_hoop_* closer to 0 MPa when compared to the SD sample.

The above analysis is further used for the stress equilibrium condition and for the *d_0_* determination formulated in the Discussion section.

### 3.5. Full-Width at Half Maximum 

**Figure 10:**
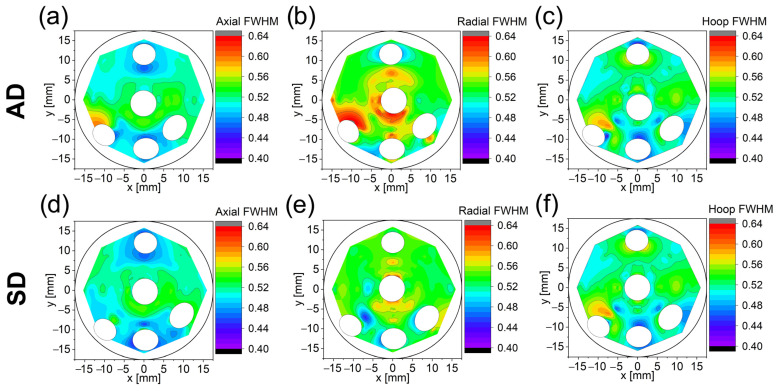
Two-dimensional plots of the FWHM [°] for axial, radial, and hoop directions for the AD (**a**–**c**) and SD (**d**–**f**) directions. The FWHM scale is indicated on the right for each plot.

The full-width at half maximum (FWHM) of the diffraction peaks was also studied considering Lines 1, Line 2, and Line 3, together with Curve 1. Two-dimensional plots have been created for both samples (see Figure 10) in axial (left), radial (center), and hoop (right) directions. The simple aim of these 2D diagrams is easier visualization and detection of possible trends.

The largest FWHM values are mainly found, for the AD sample, around the central Al wire in a radial direction in L1.3, L2.2., and L3.1. FWHM values for other directions, i.e., axial and hoop, are much lower than for the radial direction. The SD sample shows a similar trend to the AD but the effect is less pronounced and mainly distinguishable in L1.3 (radial direction). It is straightforward to infer that the FWHM is strongly affected by the processing route and by the arrangement of the Al wires, mainly at the center. The main issue is to assess if the larger FWHM in the radial direction is a consequence of the grain refinement, an increase in the microstrain, or both.

### 3.6. FEM Results

**Figure 11:**
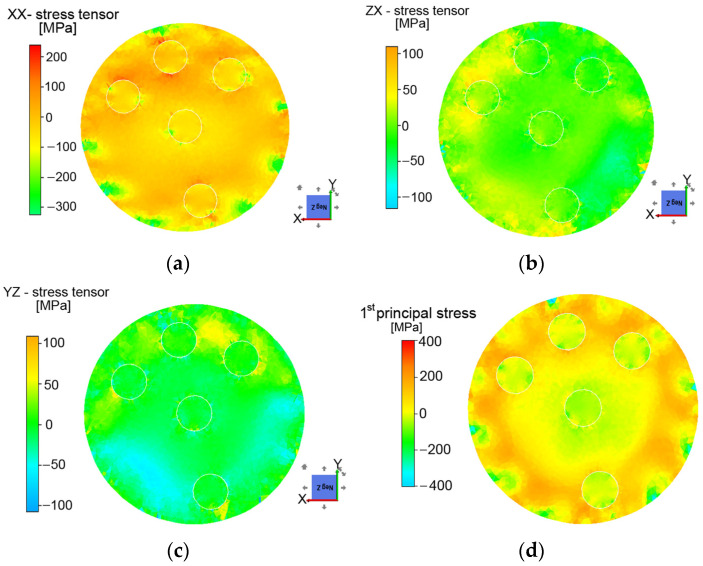
Contours of residual stresses in characteristic directions (**a**–**c**), and first principal stress (**d**).

FE analyses were used to evaluate the distribution of residual stress within the swaged composite after the first pass. As can be seen in Figure 11a–c, the vicinity of the outer Al filaments exhibited higher levels of tensile stress. The localization of the main tensile stresses is depicted in Figure 11d, which clearly shows the levels of penetration of the imposed strain from the peripheral (surface) areas of the billet into the axial ones. Such heterogeneous distribution of residual stress is caused by differences in the plastic flow, which can be variable (and is very often reversed) during the swaging process. In other words, the changes in the plastic flow and its prevailing direction are mutually connected with the character of the residual stress. In addition, note that the central Al filament was almost not affected (only slightly) by the swaging process as regards the residual stresses (i.e., the presence of residual stresses in the vicinity of the central Al filament was negligible). Moreover, the FEM results show that shear stresses are present in the specimen. Although they are smaller in magnitude than normal stresses in certain regions, they are not always negligible.

## 4. Discussion

The Monte Carlo simulation of pseudo-strain effects proved to be an efficient and rather accurate method for the recovery of intrinsic elastic strain distributions. However, the method has obvious limitations resulting from (i) the size of the sampling volume and (ii) the accuracy of the instrument and sample geometry model. Integration over the sampling volume leads unavoidably to a partial loss of information about short-range details of the strain distribution. A complex sample geometry, such as the irregular shape of Al wires after severe plastic deformation, cannot be fully described by the simulation model. It may lead to significant systematic errors in the pseudo-strain simulation. The presented experiment is an extreme case in this respect, when the pseudo-strains dominate the measured strains. The following discussion and conclusions, therefore, focus on a qualitative evaluation of observed trends, rather than on absolute values of the residual stresses. A more reliable quantitative analysis of residual stresses would be possible when future experiments were carried out with a high-flux neutron source and (ideally) a time-of-flight diffractometer, which would provide better spatial resolution and allow for easier analysis of more than three strain components at more than one diffracting plane.

A symmetric specimen (as in this investigation) and the imposed deformation constraints are deemed to develop symmetric stresses with respect to the symmetry axis (see Figure 1a,b). Nevertheless, some discrepancies have been observed in the stress differences for Line 3 (Figure 9). In the case of the SD sample, this effect is not surprising since it was already observed in previous studies for a tungsten heavy alloy [23,33]. However, when the reversal is applied (the AD sample), this effect is magnified mainly around the Al wire with no traces of symmetry except close to the sample surface.

The fact that the stress differences equal ≈0 MPa at both sides of the central Al wire for the AD sample allows us to consider the stress state around the reinforcement as hydrostatic, i.e., a full hydrostatic stress condition can be assumed in Line 3 in the Al phase. This finding enables a recalculation of the *d*_0_ in the Cu matrix with the methodology explained in [23,34]. The relation is based on the force equilibrium in the hoop direction [35] given by

(7) ∫−RRσ3CuT·dr=0

where *σ*_3_ is the total stress in the hoop direction, *R* is the bar radius, and *r* is the radial position. Since a finite number of measured points is available, the integral of Equation (7) is approximated as follows:

(8) ∑kσ3,kCuT·Δr=0

with Δ*r* being the projection length of the gauge volume along *R* and *k* representing the index of the scanned points. By combining Equations (1) and (2) with Equation (7), it is possible to recalculate the *d*_0_ for the Cu-phase as follows:

(9) $$d_{0}^{Cu}=\frac{\sum_{k}\left[A\cdot d_{3,k}^{Cu}+B\left(d_{1,k}^{Cu}+d_{2,k}^{Cu}\right)\right]\cdot \Delta r}{\left(A+2\cdot B\right)\cdot \sum_{k}\Delta r}$$

where *d_i,k_* is the interplanar distance at the measured point *k* in the direction *i*, Δ*r* has its common meaning, and *A* and *B* are factors defined as follows:

(10) $$A=\frac{E\cdot \left(1-\nu \right)}{\left(1+\nu \right)\cdot \left(1-2\nu \right)}$$

(11) $$B=\frac{E\cdot \nu }{\left(1+\nu \right)\cdot \left(1-2\nu \right)}$$

with *E* and ν having their common meanings.

Since no chemical composition variation is expected between the AD and SD samples (both samples were deformed at room temperature), the same *d*_0_ is applied for both samples.

It should be noted that residual stresses are not necessarily self-equilibrated at a local scale [36,37,38], although the gauge volume used in this study was too large to resolve type II and III stresses, i.e., only type I stresses (macrostresses) are considered. Therefore, the hypothesis of force equilibrium in the hoop direction taking into account only the macrostresses remains valid. 

**Figure 12:**
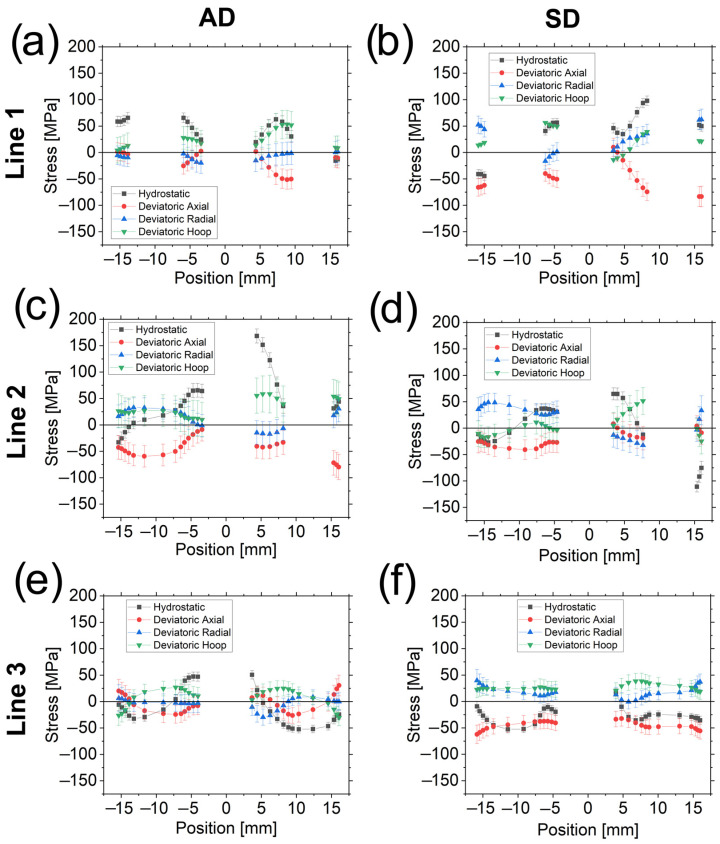
Hydrostatic and deviatoric stresses for the AD sample (**a**,**c**,**e**) and SD sample (**b**,**d**,**f**) in Line 1 (**top**), Line 2 (**center**), and Line 3 (**bottom**).

For the sake of clarity, only the hydrostatic and deviatoric stresses for the Cu-phase are shown in Figure 12. Hydrostatic and deviatoric stresses read as:

(12) $$\sigma_{H}=\frac{\sigma_{1}+\sigma_{2}+\sigma_{3}}{3}$$

(13) $$\sigma_{Di}=\sigma_{i}-\sigma_{H}$$

with *σ_H_* the hydrostatic component, *σ_Di_* the deviatoric stress in the corresponding direction *i,* and where *σ_i_* has its common meaning.

Hydrostatic stresses are typically tensile for the AD sample when close to the Al wires and compressive when far from them, with the exception of L1.4 where *σ_H_* are roughly 0 MPa (as well as the deviatoric stresses). The SD sample exhibits a similar trend, except in (i) regions between the outer surface and the Al wires (L1.1 and L2.3) and (ii) around the central Al wire in Line 3, in which *σ_H_* are compressive.

The radial *σ_D_*, mainly in regions around the Al wires, are always relatively close to zero for the AD sample. The stresses have thus a hydrostatic nature in these regions, although this behavior is not always observed in the SD sample. Moreover, the *σ_D_* in the axial direction for both samples are negative or zero (except near the outer surface for the AD sample in Line 3). More specifically, the *σ_D_* are almost negligible in L1.1 and in L1.4 for the AD sample, i.e., these regions can be considered to be only under hydrostatic stress for the former and with negligible total stresses for the latter. All *σ_D_* are zero at the interface of the Al wire in L2.3 in the SD sample.

With regard to the calculation of the stress-free reference, it was already stated that stress differences (and consequently *σ_D_*) around the central Al wire for the AD sample in Line 3 are ≈0 MPa. *σ_D_* and *σ_H_* around the Al filament, also in Line 3, for the SD sample show the same magnitude on both sides (when comparing the same stress components). It means that the hoop stresses in the Al wire can be assumed to be constant throughout this region. Therefore, Equation (8) can be used to recalculate the *d*_0_ also for this sample and this region. In fact, the difference in the lattice parameter between the two determinations is ~1.5 × 10^−6^ nm, which proves the reliability of the calculated stress-free reference.

During the rotary swaging process, the material undergoes a vortex-like flow [36]. In the case of a final 10 mm diameter rotary swaged tungsten alloy bar from a previous study [23], stresses at the center are maximum in axial (tensile) and radial (compressive) directions, and hoop stresses smaller (in magnitude) than the others. This trend is reversed when close to the outer surface and axial stresses become compressive (radial stresses approach zero to fulfill the boundary conditions). In the present study, however, we have to consider the presence of both Cu and Al phases. A bar made only of Cu should exhibit a stress profile similar to that of the tungsten alloy. The presence of the Al wires changes the deformation picture. When the Al wires are added into the Cu matrix, the latter still flows in the axial direction during the rotary swaging, but the Al–Cu interface friction partially restricts the Cu axial flow. Consequently, the axial stresses in the Cu matrix are lowered and axial deviatoric stresses become more negative in most of the regions (as observed), in contrast to the tungsten bar alloy where the axial *σ_D_* are positive.

Deviatoric stresses for the AD sample equal roughly to zero or become positive only at the Cu–Al interfaces of (i) Line 1 (except in L1.3 around the outer filament) and (ii) the central Al wire in the scanned regions where there are no other wires (L2.1 and Line 3). This is a relevant difference between the two investigated samples.

The fact that the radial *σ_D_* are close to zero for the AD sample is a consequence of both the vortex-like flow of the material and the presence of the Al filaments. When no reversal is applied between the passes, the Cu matrix flows by compressing radially (additionally to the axial flow) the Al wires. Nevertheless, a hoop component exists, which causes the stresses within the Al to be not purely hydrostatic. The significant effect of the hoop component is clearly proved by the elliptical shape of the Al wires placed near the surface (see Figure 1a,b) [39]. When the reversal is applied after the first pass, the material experiences the vortex-like flow again, although with one main difference: the hoop flow is in the other direction, with the Cu matrix recovering its previous position around the Al wires, and the hoop and, mainly, the radial stresses becoming more hydrostatic, as it would be expected if external hydrostatic forces were exerted. This effect can explain why the stresses become roughly hydrostatic around the wires in Line 1 (except in L1.3 around the outer filament), L2.1, L3.1, and L.3.2.

In addition to the calculated hydrostatic and deviatoric stresses, a direct consequence is that the stresses in the central Al wire are not fully hydrostatic over the entire Al cross-section, but vary depending on the scanned line. However, in the particular case of Line 3, it can be estimated to be approximately ≈47 MPa for the AD sample, i.e., equal to the magnitude of the hydrostatic stress in the Cu matrix around the Al.

Given the complexity of the residual stress profile, an easy indicator of the overall stress state in the material after the rotary swaging processing is the stresses calculated with the von Mises formula. The shear stresses could not be determined due to the limited neutron flux and available beam time (although their presence can be expected in some regions due to the die–sample friction), but they are assumed to be lower (as it can be inferred from the FEM simulation) than the normal stresses. By neglecting the shear stresses, the von Mises relation only accounts for the normal stress differences (or, equivalently, the deviatoric stress differences). The simplified relation reads as follows:

(14) $$\sigma_{VM}=\sqrt{\frac{1}{2}\left[{\left(\sigma_{axial}-\sigma_{radial}\right)}^{2}+{\left(\sigma_{axial}-\sigma_{hoop}\right)}^{2}+{\left(\sigma_{radial}-\sigma_{hoop}\right)}^{2}\right]}$$

It is important to remark that the directions in which the stresses have been calculated are not necessarily principal. Consequently, the previous relation represents a first approximation and a lower limit of the actual ones.

**Figure 13:**
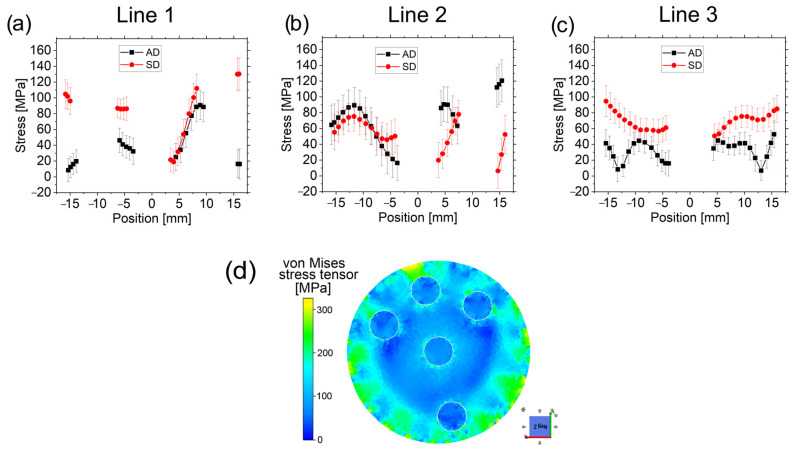
Stresses calculated with the VM relation for the AD and SD samples in the scanned lines 1 (**a**), 2 (**b**), and 3 (**c**). The VM stress distribution obtained from the FE analysis after the first pass is shown in (**d**).

Von Mises (VM) stresses are presented in Figure 13. In Line 1, VM stresses for the AD sample are much lower in the same region than for the SD sample (except in L1.3), which is a consequence of a more hydrostatic nature of the stresses, according to previous observations (Figure 12). The line within the region of a high density of Al wires, i.e., L.1.3, exhibits practically a full coincidence between the two samples, starting with low stresses at the center and increasing their magnitude as the distance from the center increases.

VM stresses in Line 2 are similar in L2.1 for both samples close to the outer surface but show a different trend in L2.2 and L2.3 with higher stresses for the AD sample, as opposed to what is observed in Line 1.

Line 3 shows that the SD sample exhibits higher VM stresses. They can also be considered symmetric except around the central Al wire, where a small deviation is observed. This symmetric trend of VM stresses (although the total stresses are not symmetric) has been previously observed in a tungsten heavy alloy studied in [33], and it proves the reliability of the pseudo-strain correction.

Understanding the deformation process in the area with the highest wire density (L1.3) and around it (L2.3) is not a simple task. We can hypothesize that the plastic flow of the Cu matrix (mainly in the hoop direction) in L1.3 and L2.2 is different to that in L1.4 and L.2.3. The proximity of the Al wires could form a “barrier” due to an Al–Cu friction in axial and hoop directions and the stress field around them, which would lead to different plasticity rates (as they are decoupled) in the inner and the outer part of the Cu matrix surrounding the peripheral wires. When comparing the VM stresses in L2.2 around the peripheral wire with those in L3.2 in the outer surface (for the same sample), no differences are observed. The former region (L2.2) could be assimilated into the outer region. However, it is still a not well-understood process that requires additional studies.

Stresses calculated with the von Mises relation can be compared with the yield stress *σ_y_* of the Cu matrix (130 MPa). The maximum VM stresses observed are for the SD sample in L1.4 with ≈130 MPa and in L2.3 for the AD sample with ≈120 MPa, i.e., equal to or lower than the 130 MPa yield stress. It helps to validate the reliability of the calculations and suggests that in these regions (L1.4 for the SD sample and L2.3 for the AD sample), the shear stresses can be considered negligible.

As general remarks, it can be stated that:

- The reversal of the bar direction contributes to lowering the deviatoric stress components in Line 1 (except in L1.3 near the outer Al wire). It seems to be advantageous in order to delay material plastification and failure;
- Within the region with a high density of Al filaments, the reversal between the passes does not change significantly the overall state for both samples;
- The line delimiting one side of the high-density Al wire region (L2.3) for the AD sample is prone to failure as well as the L1.4 for the SD;
- The filament-free region exhibits a roughly symmetric behavior but with lower VM stresses for the AD sample.

The FE analysis also provides VM stresses but also accounts for the shear stresses (Figure 13d). It should also be taken into account that neutron measurements required the use of large gauge volumes, which smooth the obtained stresses. Therefore, the VM stresses calculated with the FEM simulation are expected to be larger than those experimentally measured by neutron diffraction. Although the simulation shows the VM stress distribution after the first pass only, a coincidence in the trend for the Cu matrix can be observed with the neutron measurements (mainly for the SD sample since there is no reversal causing the more significant change in the stress distribution). The main coincidences are that (i) VM stresses near the outer surface are at a maximum in the scanned lines and lower when close to the central filament, and (ii) the simulation captures the VM stress increase around the inner side of the outer filaments (L1.3, and L2.2 segments).

The FWHM comprises information about the size of the coherently scattering domains [40] (hereafter for the sake of brevity referred to as “crystallite”) and the microstrain. During the rotary swaging, the material flows plastically in axial and hoop directions in a vortex-like flow [39], and the presence of dynamic recovery or dynamic recrystallization has already been stated in some works [41], although for bars with smaller final diameters. It is plausible to hypothesize that a restoration phenomenon occurs in the bars during the plastic deformation process. In addition, the initial microstructure exhibited annealing twins (see Figure 1c), which also affects the FWHM. The dislocation rearrangement/annihilation typically involves a reduction in the microstrain and an increase in the crystallite size, i.e., the FWHM decreases. The restoration phenomenon is expected to occur equally in all directions (affecting equally the annealing twins), thus giving raise to similar FWHM values in all directions. Therefore, the only plausible parameters that could differ between different directions are the microstresses (or Type II and III stresses) with some oriented and more pronounced elastic mismatch in the radial direction between the Cu matrix and the Al filament (note that the largest variation occurs around or near the Al wires). The main argument to support this hypothesis is that the gauge volume was too large to resolve microstresses, so they only visibly contributed to the peak broadening [24].

**Figure 14:**
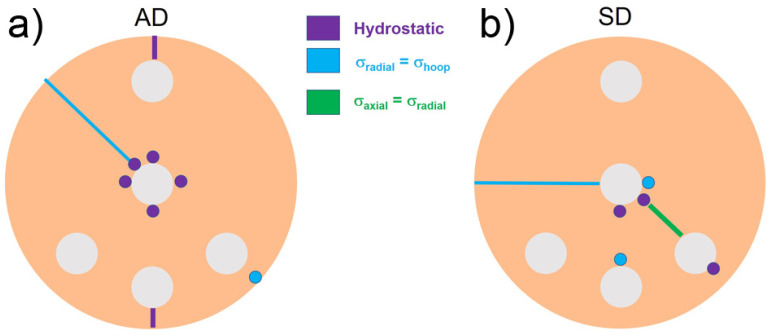
Stress states developed in the (**a**) AD and (**b**) SD samples. The biaxial stress states are also indicated.

Finally, in order to get a better overview of the complex stress states developed in different regions of the samples, Figure 14 summarizes the previous stress configuration results.

## 5. Conclusions

The following conclusions can be drawn from the present work:

- Hydrostatic stresses for the AD sample tend to be tensile in regions within the Al wires or surrounding them. Only far from the Al wires do hydrostatic stresses become compressive. The SD specimen exhibits more regions with compressive hydrostatic stresses;
- Axial deviatoric stresses are zero or compressive in most of the regions for both samples;
- The reversal of the bar direction provokes a lowering of the deviatoric stresses in the regions far from the Al wires and around the low Al-wire density regions where the stresses tend to be hydrostatic;
- The reversal of the bar direction slightly changes the overall state within the region of the high density of Al wires. The stresses calculated with the von Mises relation are at a maximum when close to the outer Al wire for both samples. In regions without Al wires, the reversal of the bar seems to be advantageous to avoid a possible plastification;
- Von Mises stresses calculated with the FEM simulations are higher than those measured with neutron diffraction; among other reasons, the presence of shear stresses may be possible;
- It was found that the reversal of the bar direction seems to be advantageous for the component properties. The reversal lowers the deviatoric stress components in a significantly larger part of the volume examined, although not in all the scanned segments;
- FEM shows that there can be a large variability of residual stress along the circumference. The residual stresses calculated using the von Mises relation from the neutron diffraction data can be approaching the yield stress of the Cu matrix near the outer surface of the component. It occurs regardless of the applied deformation mode (reversal, no reversal), although this effect seems to be more pronounced for the no-reversal component. Further optimization should aim at setting the processing parameters to still lower the stresses near the surface.
- The full-width-at-half-maximum of diffraction peaks is largest in the radial direction near the central Al wire in the region with a higher density of filaments and may initially be attributed to a predominance of microstresses in the radial direction;
- A novel evaluation procedure was successfully used for pseudo-strain treatment.

## Figures and Tables

**Table 1:** Values of parameters at 25 °C used in the simulation.

Property	Unit	Cu	Al
Young modulus	(GPa)	111	72
Poisson ratio	-	0.3	0.3
Density	(g.cm^−3^)	8.96	2.80
Specific heat	(J.kg^−1^.K^−1^)	398	1230
Emissivity	-	0.7	0.03
Thermal expansion	(K^−1^)	1.7 × 10^−5^	2.4 × 10^−5^
Thermal conductivity	(W/(m.K))	394	250

## Data Availability

The data that support the findings of this study are available from the corresponding author upon reasonable request.
